# Connexin and pannexin channels in cancer

**DOI:** 10.1186/s12860-016-0094-8

**Published:** 2016-05-24

**Authors:** Jean X. Jiang, Silvia Penuela

**Affiliations:** Department of Biochemistry, University of Texas Health Science Center, San Antonio, TX 78229 USA; Department of Anatomy and Cell Biology, University of Western Ontario, London, Ontario N6A5C1 Canada

## Abstract

Communication among cells via direct cell-cell contact by connexin gap junctions, or between cell and extracellular environment via pannexin channels or connexin hemichannels, is a key factor in cell function and tissue homeostasis. Upon malignant transformation in different cancer types, the dysregulation of these connexin and pannexin channels and their effect in cellular communication, can either enhance or suppress tumorigenesis and metastasis. In this review, we will highlight the latest reports on the role of the well characterized connexin family and its ability to form gap junctions and hemichannels in cancer. We will also introduce the more recently discovered family of pannexin channels and our current knowledge about their involvement in cancer progression.

## Background

Intercellular communication between cells is realized through gap junction channels formed by connexins (Cx). In the last decade or so, connexin hemichannels were identified, which mediate the communication between intracellular and the extracellular microenvironment [1, 2]. It is well documented that gap junctional intercellular communication is important in mediating normal cell growth, differentiation, and development. The roles of non-coupling hemichannels started to emerge [3], mostly resulting in the disruption of normal homeostasis, although under mechanical stimulation they mediate the release of important anabolic factors in bone cells [4, 5]. Among the various types of connexins, mutations of connexins or loss of functional channels are implicated in many diseases and disorders, including congenital deafness, skin disorders, cataracts and cancers [6, 7].

Pannexin channels on the other hand (Panx1, 2 and 3), are a recently discovered family [8] of single membrane channel-forming glycoproteins expressed in many organs of the human body [9–11]. Pannexins were first discovered due to their limited sequence homology to innexins [9] (invertebrate gap junctions), and were initially proposed as a new family of mammalian gap junction proteins, complementary to connexins [12]. Although their capacity to form gap junctions remains controversial [13, 14], we and others have shown that their main function is to form large-pore single membrane channels for the release of ATP and other metabolites important for cellular communication and autocrine/paracrine signaling [10, 15–19]. Panx1 has been reported to be ubiquitously expressed in most mammalian cells and tissues [9, 10]. Panx2 is expressed in neurons of the central nervous system and many other tissues as was recently reported [20]. Panx2 has been linked to modulation of neuronal commitment, being differentially expressed in postnatal neural progenitor cells and mature neurons [21]. Panx3 is expressed mostly in keratinocytes, chondrocytes and osteoblasts where it regulates cell proliferation and differentiation [9, 18, 19, 22, 23].

Panx1 represents the best characterized member of the family. Panx1 is N-glycosylated at residue N254 in the second extracellular loop (EL2) [10, 24], and this modification regulates its localization in the cell and the ability of Panx1 to intermix with other pannexins [16]. Six subunits of Panx1 can oligomerize into a hexamer [24] to form a channel that is thought to be gated closed by its carboxyl terminal tail (CT), interacting with the intracellular loop (IL) in a ball-and-chain fashion [25, 26]. The regulation of Panx1 channel opening is not well understood, but individual cysteine and tyrosine residues in the intracellular loop of the protein appear to be critical for Panx1 channel function [27–30]. The large-pore Panx1 channel can allow passage of molecules of ~1 kDa when open (500 pS), and it can be activated by voltage, high extracellular K^+^, high intracellular Ca^2+^ and mechano-sensitive stimuli among others [17, 31]. Panx1 has been proposed to have a role in inflammation [32–34]. Additionally, Panx1 channels can be irreversibly opened by caspase cleavage of the CT, activating the release of ATP as a ‘find-me’ signal for monocyte recruitment and clearance of apoptotic lymphocytes [15].

In the current review, we will highlight the most recent literature on the role of connexins in cancer, and introduce the more recently discovered pannexin channels and their proposed function in different cancer types.

## Connexins and cancer

Cancer was one of the first pathologies found to be associated with gap junction channel impairment, and connexins have long been shown to possess tumor suppressive function [35]. Cx43 as well as other members of the connexin family such as Cx26 and Cx32 have been demonstrated to inhibit primary tumor cell progression when expressed in tumor cells [36–38]. However, connexin expression has been positively correlated with more invasive cancers and metastases [39, 40]. These data suggest a more complex, cancer stage-specific function of connexins in cancer progression and metastasis. Connexin expression is also reported to suppress cancer behavior and this function is independent of their roles in forming gap junction and hemichannels [41]. Many review papers in recent years cover the topics related to connexin and connexin-forming channels in cell cycle progression and checkpoint control, cancer cell proliferation and differentiation, cancer microenvironment, tissue invasion and metastasis, and various cancer pathologies and therapeutic potentials (for reviews, see [7, 39, 40, 42–52]). In this review, we primarily focus on studies published in the last couple of years (Table 1), particularly concentrating on the underlying mechanisms related to various states of cancer development and their potential applications in cancer therapeutics.

**Table 1:** Summary of the most recent progress in our understanding of connexin subtypes in growth and metastasis of various cancers

Connexin	Cancer type	Description	Reference
Cx43	Breast	Different effects on MCF-7 and MDA-MB231 in 2-D and 3-D cell cultures	[65]
Cx43	Breast	Peptide ACT1 increase GJ and impairs cell proliferation/survival	[116]
Cx43	Breast	CM from breast cancer cells decreases Cx43 gap junctions	[96]
Cx43	Gastric cancer cells	Increase in diapedesis and transmigration through intercellular space between mesothelial cells	[90]
Cx43	Mammalian cancer stem-like cells	miRNA-206 represses pro-apoptotic PDCD4 and Cx43	[76]
Cx43	Prostate	With Snail-1 to repress E-cadherin and promotes EMT, mobility and invasiveness	[88]
Cx43	Prostate	Cx43 knockdown, not gap junctions inhibits migration/invasion of four prostate cancer cell lines	[98]
Cx43	Osteosarcoma	miRNA-23a suppresses Cx43	[77]
Cx43	Neuroblastoma & glioma, leukemia	A direct link of Cx43 and CCN in anti-proliferative function	[67]
Cx43	C6 glioma	Cx43 antibody in rats increases survival and even more with radiotherapy	[122]
Cx43	Glioma	Cx43 increases resistance to TMZ via mitochondrial apoptosis pathway	[136]
Cx43	Astrocytomas & glioblastomas	Increased Cx43 expression in human tumors	[125]
Cx43	Brain metastasis	GJ with endothelial cells to promote metastatic lesion	[86]
Cx43	Lung adenocarcinoma	Increased incidence by DMBA in Cx43 heterozygous knockout mice	[62]
Cx43	Non-small Lung cancer	Cx43 reduction as a marker for cisplatin-chemotherapy	[132]
Cx43	Non-small Lung cancer	Cx43 overexpression reverses EMT and cisplatin resistance and deletion has opposing effects	[134]
Cx43	Glioblastoma multiforme (GBM)	HCMV reduces Cx43 and gap junctions	[64]
Cx43	GBM	Cx43 increased in TMZ-resistant cells by EGFR signaling	[135]
Cx43	Mesothelioma	Increases sensitivity to sunitinib via interaction with Bax	[109]
Cx43	Oral carcinoma	Cx43 correlates as a prognostic biomarker	[130]
Cx43	HBV hepatocellular carcinoma	Cx43 expression correlates with early tumor recurrence and survival	[133]
Cx43	Ras-transformed NIH-3 T3 cell	Cx43 5′ UTP active in transformed cell and 3′ UTP active in normal cell	[72]
Cx32	Prostate	Increased by Vit.D3 and sensitizes cells to growth inhibition by Vit.D3	[63]
Cx32	Pancreatic and prostate cancer	Cx32 C-terminus required for growth and stability of gap junctions	[68]
Cx37	Gastric tumors	Cx37 siRNA negatively correlated with cancer development and promotes apoptosis	[124]
Cx26	Cervical cancer-HeLa	Redistribute cAMP and blocks cell cycle progression	[69]
Cx46 & Cx43	Glioblastoma cancer stem cell	Cx46, not Cx43 is detected; During differentiation, Cx46 reduced and Cx43 increased	[126]
Cx30 & Cx43	Breast	Cx30 deletion and mutation in human tumors; Cx30/Cx43 as potential prognostic markers	[128]

## Connexin channels in cell cycle progression, cell proliferation and primary cancer growth

Loewenstein and Kanno first postulated that liver cancer cells were different from normal liver cells in that they lacked intercellular communication [53]. Since then, there has been numerous studies dedicated to uncover the role of gap junctions and connexins in tumor progression (reviewed by [6, 54, 55]). Reduced expression of connexins and gap junctional communication has been shown in many tumor types. Clinical studies also report that deficient or abnormal connexins are frequently found in tumor tissues and cell lines, such as breast cancer, prostate cancer, lung cancer, and many other cancer types [7]. For example, Cx43 was found to be reduced in breast carcinoma cells obtained from the primary tumors of breast cancer patients [56]. In some cases, decreased expression of connexins even occurs in precancerous lesions; Cx43 was found to be highly reduced in cervical dysplasia, an early stage in malignant transformation [57].

Many studies demonstrate that cancer cells lose their capability of communication through gap junctions, leading to the overall assumption that cancer cells do not communicate. However, the situation is more complicated than originally thought. Some cells completely lack cell coupling, whereas others exhibit a slight decrease compared to their normal counterparts [35]. While it is true that the tumorigenic behavior of cells is not always correlated with a complete lack of cell-cell communication, the forced inhibition of connexins and cell coupling, such as knocking down connexin expression, has enhanced the tumorigenicity of carcinoma cells. In vivo studies demonstrate the tumor suppressive roles of Cx43 and Cx32, in which mice with decreased expression of Cx43 or Cx32 exhibit increased carcinogen-induced tumor growth in comparison to control wild-type mice, represented by an increase in number and size of tumor nodules in the lung [58, 59]. A recent study showed that ectopic expression of Cx43, not Cx26, suppresses cell proliferation and anchorage-independent growth, and also reduces size of melanomas when grown in the chicken chorioallantoic membrane, a unique ex vivo system to study cancer growth [60]. Additionally, inhibition of Cx43 gap junctions accelerates proliferation of four different melanoma cell lines whereas as expected, increased coupling through overexpression of Cx43 reduces cell growth and melanoma tumor growth in vivo [61]. Increased incidence of lung adenocarcinomas induced by 7,12-dimethylbenzanthracene (DMBA), a carcinogen, was observed in Cx43 heterozygous knockout mice compared to wild-type control mice [62]. Active vitamin D3, a chemo-preventive agent, increases expression and assembly of Cx32 and prevents androgen-induced degradation of Cx32 in human prostate cancer cells [63]. Additionally, Cx32 gap junctions sensitize cells to the growth inhibitory effect of vitamin D3. Human cytomegalovirus (HCMV) is highly prevalent in glioblastoma multiforme (GBM) and in other tumors, and HCMV downregulates Cx43 protein and disrupts gap junctional coupling, which is thought to be a mechanism contributing to gliomagenesis [64]. Suppression of tumor growth also depends upon cancer cell types and context. For example, Cx26 and Cx43 overexpression in the breast cancer cell line, MDA-MB-231, inhibited tumor growth in a three-dimensional (3D) environment [40]. Cx43 exerts different degrees of inhibitory effects on two breast adenocarcinoma cells, ER+ MCF-7 and triple negative MDA-MB-231 cells. Interestingly, the effect on 2D and 3D cultures of identical cancer cell type is also different [65].

The major effect of connexins and gap junctions on suppression of primary cancer growth is a result of inhibition of cell cycle progression; however, until now limited knowledge is gained regarding direct molecular mechanisms governing the cell cycle cell events in cancer cells. An earlier evidence of the effect of Cx43 on cell cycle is exemplified in C6 glioma cells, where the restoration of Cx43 inhibits their progression to the S phase of the cell cycle, owing in part to a reduction of cyclin E expression and thus, the rate of proliferation [66]. Interestingly, the restoration of Cx43 expression in glioma cells diminishes c-Src activity. Additionally, a Cx43 mutant lacking its c-Src phosphorylation sites is not able to inhibit the c-Src activity and does not exhibit decreased glioma proliferation. This suggests that c-Src phosphorylation on the C-terminal tail of Cx43 is involved in the anti-proliferative effects. This data suggests that Cx43 and c-Src are mutually regulated by a reciprocal activation/inactivation loop via protein phosphorylation. Gellhaus et al. [67] reported a direct link between Cx43 and the growth regulator protein CCN3, which is involved in the anti-proliferative activity of neuroblastoma, glioma, and chronic myeloid leukemia. FRET analysis and co-immunoprecipitation results establish an interaction between CCN3 and the C-terminus of Cx43 in human embryonic kidney epithelial (293 T) cells. Several variants of Cx43 with amino acid deletions were generated, and the interaction of the Cx43 variants with CCN3 was analyzed using FRET analysis. A 3D model of the CCN3/Cx43 interaction complex was constructed, and CCN3 specifically binds to the C-terminus of Cx43. The ability of Cx43 to reduce the growth of 293 T cells was facilitated by its binding with CCN3, leading to an increase in endogenous CCN3. C-terminus of Cx32 is shown to be not required for initiation of the assembly of gap junctions, but for the further growth and stability in human pancreatic and prostatic cancer cells [68]. A recent study suggests that the role of Cx26, unlike Cx43 and Cx32, in suppression of cervical cancer derived HeLa cell growth likely results from a redistribution of cAMP throughout the cell population. This function eliminates the cell cycle oscillations due to higher concentration of cAMP required for cell cycle progression [69]. Tumor cells in G2/M phases of cell cycle are associated with low-dose hyper-radiosensitivity (HRS). A recent study reported that Cx43 downregulation, a feature of the cell at G2M phase, somehow renders tumor cells hypersensitive to low-dose radiation occurring at G2M phase is a major cause of HRS in multiple tumor cells in vitro [70]. The downreguation of cyclin-dependent kinase 6 by miRNA-124-3p transferred between glioblastoma cells via gap junctions promotes the anti-proliferative effect [71]. The different mechanism involving post-transcriptional regulation of Cx43 is recently reported in H-Ras-transformed cells compared to non-transformed parental cells; 5′untranslated region (UTR) is active in Ras-transferred NIH-3 T3 cells while the 3′UTR is active in normal, non-transformed NIH-3 T3 cell and a regulatory element in the 3′UTR was identified [72]. This study suggests specific regulatory mechanisms in regulation of connexin expression in cancer cells.

More interestingly, recent studies show that epigenetic processes play an essential role in regulating connexin gene expression which includes histone modifications, DNA methylation and microRNA (see review [73, 74]) A study shows that downregulation of the microRNA miR218 is associated with nasopharyngeal carcinoma (NPC), an Epstein-Barr virus-associated malignancy [75]. Suppression of miR-218 is related to epigenetic silencing of SLIT2 and SLIT3, ligands of ROBO receptors involved in tumor angiogenesis. Moreover, this microRNA directly interacts with 3′-UTR of Cx43. MicroRNA-206 in mammary cancer stem-like cells (McCSCs) mediates Zinc-finger transcription factor Kruppel-like factor 4 (KLF4) pro-survival signaling through repression of the pro-apoptotic molecules programmed cell death 4 (PDCD4) and Cx43 [76]. Another study shows miRNA-23a suppresses Cx43 and impairs osteoblast differentiation of an osteosarcoma cell line [77]. On the other hand, gap junction communication can mediate the transfer of some microRNAs between cells. A recent study reported by Suzhi et al. that gap junctions mediates transport of miRNA-124-3p between cells and enhances the anti-proliferative effect of this miRNA in two glioblastoma cells [71]. This is a current active research area and more studies are expected revealing the mechanistic roles of connexins and gap junctions in facilitating the function of miRNAs.

## Connexin channels in metastasized cancer cells and cancer microenvironment

The major questions remain whether connexins are tumor suppressors or tumor promoters; the situation is much more complex, and no clear answer can be made thus far. There are many points to consider. The effect of connexins on tumor progression and behavior depends largely on endogenous connexin expressed or overexpression of exogenous connexins on the particular cells and tissue examined. Furthermore, the inconsistent evidence of connexins’ role in cancer reflects the complexity of developmental stages of cancer progression and metastasis. The existence of a correlation between connexin expression and the invasive potential, as opposed to the lack of connexin expression in the earlier proliferation period of cancer progression points to a specific function of connexins during cancer development and metastasis. For example, Cx43 has a multifaceted function depending upon the stages of cancers; with low expression in primary tumor and high expression in metastasized tumor. Cx43 is involved in neural crest cell migration as a part of normal development and that impaired gap junction communication leads to reduced neural crest cell migration [78]. Consistently, in invasive cancers, such as gliomas, increased Cx43 expression has also been demonstrated to enhance the cell mobility and invasion [79], which is contrary to the involvement of Cx43 expression in tumor growth. In human breast cancer, an earlier study shows that both Cx26 and Cx43 are upregulated in invasive lesions compared to normal breast cells and cells from non-invasive lesions, although Cx26 is localized to the cytoplasm, whereas Cx43 was to intercellular locations [80]. Clinically, increased expression of Cx26 and Cx43 has been discovered in lymph node metastases of breast cancer, as opposed to primary breast tumors with decreased expression of connexins [81]. A narrow range of low doses of γ-radiation increases Cx43 expression, activation of p38 MAPK/p21 and glioma cell migration, but has no effect on cell proliferation, and the activation of p38 is inhibited by Cx43 knockdown, suggesting Cx43 is an important mediator in MAPK activation and radiation-induced secondary malignancies and metastasis [82].

In contrast to the inhibitory effect of connexins in primary tumors, a promoting effect has been found in metastatic cancers. Some studies have found that increased gap junction coupling and Cx43 expression contribute to breast cancer adhesion and migration [83]. The establishment of cell-cell communication could be beneficial in the diapedesis of breast tumor cells during extravasation through the endothelium. This has been observed in breast cancer cells, which establish heterotypic gap junctions with endothelial cells [84]. Furthermore, overexpression of Cx43 enhances the adhesion of the cancer cells to the lung endothelial cells [85]. Breast cancer cells also use Cx43 to initiate brain metastatic lesions by forming gap junctions with the endothelial cells in the vasculature, whereas knocking down connexins by RNAi or pharmacological methods inhibited brain colonization [86]. Plante et al. showed that in a mouse model expressing mutant Cx43 (G60S mice), there was a delayed onset of palpable mammary gland tumours compared to controls, but an increase in lung metastases, highlighting the different effects of Cx43 in primary and secondary tumours [87]. Cx43 levels are well correlated with Snail-1, a transcription factor which represses E-cadherin and promotes EMT in prostate cancer cells; high expression of these two molecules is accompanied high mobility and elasticity of cancer cells, and the invasiveness features of cancer cells [88].

Cancer metastases could also be promoted with the aid of heterotypic gap junctions formed between the migrating cancer cells and the target tissue cells. For example, the formation of heterotypic gap junctions between a human breast cancer cell line and an osteoblast cell line has been demonstrated, where the degree of heterotypic gap junctions relative to homotypic ones correlated with breast cancer cell metastatic potential [89]. This data may implicate a mechanism occurring in breast cancer metastasis to the bone. Heterotypic gap junction communication between gastric cancer cells and mesothelial cells appears to play an active role in peritoneal metastasis. Human gastric cancer cells overexpressing wild-type and gap junction-impaired Cx43 mutant were co-cultured with human peritoneal mesothelial cells, and significant increase in diapedesis and transmigration of cancer cells through intercellular space between the mesothelial cells was shown in wild-type Cx43, not in mutant, expressed cells [90]. The lymphatic vasculature is a known conduit for metastasis and increased interaction between tumor cells and lymphatic endothelial cells was thought to play a key role. Adrenomedullin, a peptide hormone promotes this interaction and increases gap junction coupling [91]. Blockade of this heterotypical communication prevents the tumor cells to migrate through the lymphatic monolayer. A long-range gap junction signaling model in control of cancer growth from normal host cells was proposed by which disruption of gap junction coupling farther away from oncogene-expressing cells has profound effect in suppression of tumor incidence [92].

The tumor suppressive effect of connexins has also been demonstrated in several metastatic cancers. In a highly metastatic lung cancer cell line, Cx43 expression was undetected, whereas transfection of Cx43 inhibited the tumorigenicity of the cells [93]. Similarly, increased human breast cancer metastatic potential was found to correlate with decreased Cx43 gene expression and decreased gap junction communication [94, 95].

Tumor microenvironment also impacts connexins and gap junctions in normal cells. It is known that interaction with mural cells causes endothelial cells of blood vessels to become quiescent. However, conditioned media collected from breast cancer cells diminishes gap junctional coupling between mural cells and endothelial cells, and downregulates Cx43 in mural cells [96]. Additionally, tumor implantation also significantly increased tumor vascularization in heterozygous Cx43 knockout mice.

## Channel-independent role of connexins in primary and metastasized cancer

Several published studies suggest that connexin proteins influence cell cycle and signaling independently of their roles in forming gap junctions or hemichannels through its impact on cell cycle and signaling [97]. A recent study shows that Cx43 expression in four prostate cancer cells lines is well correlated with metastatic potential; however, only knocking down Cx43, not inhibition of gap junction channels, decreases cell migration and invasion [98]. Connexin proteins are reported to affect the production and activity of many cell cycle regulators, including p27Kip1, cyclin A, cyclin D1, cyclin D2, ERK1/2, Src, and FGF1 [99–104]. Moreover, the cytoplasmic C-terminal tail domain of Cx43 alone has been shown just as effective as the full protein in suppressing neuroblastoma progression possibly through its effects on Src molecule [105]. Another example of the channel-independent involvement of connexins in tumorigenic cell signaling and cell cycle was reported by Langlois et al. [106]. In this study, they examined the effects of Cx43 interaction with the skin tumor suppressor caveolin-1 (Cav-1) in rat epidermal keratinocytes (REK). REK cells with reduced Cx43 expression exhibited increased epithelial-mesenchymal transition (EMT) as well as more invasive features than the control cells. Blocking the gap junctions with carbenoxolone does not affect the invasive behavior; thus, connexin channels may not be involved although carbenoxolone is known to block pannexin channels as well [107]. This is likely to be caused by the interaction between the Cx43 C-terminal tail domain and this interaction is implied to be crucial in suppression of keratinocyte transformation and invasion. This was also confirmed in vivo, where the co-localization of Cx43 and Cav-1 was reduced in epidermal squamous cell carcinoma cells. The channel-independent role of connexins has also been implicated in the regulation of migration and proliferation through its interactions with the actin cytoskeleton. The effects of Cx43 in mediating the regulation of the actin cytoskeleton was studied in human glioma cells [108]. Two truncated constructs of Cx43: one lacking the C-terminal tail domain and one lacking the entire transmembrane domain are used, and there is a similar reduction in the proliferation of glioma cells regardless of whether the truncated or full length versions of Cx43 are used. Truncated Cx43 does not alter gap junction coupling and furthermore, Cx43 C-terminal tail domain is sufficient to promote migration of glioma cells. The C-terminal tail domain is associated with a lamellipodia-type migration, which implies the involvement of Cx43 in the regulation of the actin cytoskeleton. Additionally, cell mobility of glioma cells is not affected by gap junction inhibitors, whereas only cells overexpressing full-length Cx43, not truncated Cx43 without the C-terminal tail domain increases mobility [79]. A recent study showed that Cx43 increased sensitivity to sunitinib-induced cytotoxicity in malignant mesothelioma cells, and this effect is independent of gap junctions and is likely exerted through its direct interaction with apoptotic factor Bax [109]. Cx37 suppresses cancer cell growth and six extracellular cysteine residues involved in gap junctional docking were individually mutated. Of six mutants, three traffic to the plasma membrane as wild-type Cx37, but none form functional gap junctions and two form hemichannels. However, none of these mutants affect insulinoma (Rin) cell proliferation [110]. Moreover, by using domain swapping chimeric mutants, C-terminus and pore-forming domains are found to be essential to the role of Cx37 in growth suppression of Rin cells [111].

The effect of Cx43 expression on the regulation of cell cycle was investigated in HeLa cells transfected with Cx43 and primary human foreskin fibroblasts (HFF) predominantly expressing Cx43 [112]. 18-α-glycyrrhetinic acid (GA) was used to reduce gap junctional coupling but not affect Cx43 expression, and sodium butyrate (NaBu) or anti-arrhythmic peptide (AAP10) were used to enhance Cx43 expression in the cells. Using time-lapse microscopy, it was discovered that the increase in Cx43 expression levels delayed mitotic durations, which corresponded with an accumulation of cells in the G1 phase, leading to a reduction in cell proliferation. This was linked to the increased expression of p21waf1/cip1, which is a cell cycle inhibitor. These effects were unrelated to Cx43 channel function. Overall, it is suggested that the upregulation of Cx43 expression delays the rate of cell cycle through the delay of G1, thereby increasing time between mitotic cycles; however, the underlying mechanism remains largely elusive.

## Connexin channels in clinical studies and potential cancer therapeutics

There are several plausible strategies in the development of potential therapeutics. The initial attempts to reverse the lack of gap junctional communication known in tumorigenic cells were accomplished through chemical treatments, including retinoids, vitamin D, carotenoids, and cAMP [113]. By taking an advantage of inclusive gap junction communication only between tumor cells, not with surrounding cells, tumor cells were loaded with Lucifer yellow (LY) and implanted into tumor in situ. By irradiation with blue light, only tumor cells containing Lucifer yellow died, not surrounding cells without LY [114]. Furthermore, these experiments of forced re-induction of gap junction communication between a tumor cell and a non-tumorigenic cell were able to prevent the growth of transformed cells [115]. Cx43 gap junctions are reduced in breast cancer and effects have been made to restore gap junction activity in these cells. A therapeutic peptide ACT1 enhances Cx43 gap junction function and impairs proliferation or survival of breast cancer cells [116]. Additionally, combination of ACT1 with tamoxifen or lapatinib augments the effects of these drugs on ER+ or Her2+ breast cancer cells, respectively. However, it is important to note that the recovery of gap junction coupling does not always automatically entail normalization of the tumor cells. Carbon monoxide is emerging as a promising molecule to treat several diseases including cancer. It can directly or indirectly inhibit the function of connexin hemichannels [117, 118] although the role of hemichannels in cancer cells is yet to be uncovered. *Ganoderma lucidum*, an herbal mushroom increases Cx43 expression and inhibits growth of human ovarian cancer cells, and this growth inhibition was abrogated by knocking down Cx43 gene [119]. The bioactive substance sulforaphane inhibits cancer stem cells in pancreatic ductal adenocarcinoma through the increased Cx43 and E-cadherin expression, altered activation of several kinases and enhanced gap junction channels [120]. Several lipophilic statins are suggested as antineoplastic agents. A recent paper shows that the effect of simvastatin is mediated by Cx43 gap junctions via inhibition of phosphorylation of Cx43 by PKC [121]. All these studies implicate Cx43 as a potential therapeutic target. Indeed, Cx43 antibody against extracellular loop domain was tested in Wistar rats with induced C6 glioma [122]. Treatment with this antibody increased median survival to 39.5 days compared to 28 days without treatment, and combined treatment with radiotherapy increased survival to 60 days. Antibody against Cx43 E2 domain blocks Cx43 hemichannels [123]; thus Cx43 hemichannels is likely involved in the growth of glioma cells. In addition to Cx43, small interfering RNA (siRNA) was used to knockdown Cx37 expression in subcutaneous gastric tumors in mice [124]. Their results show that the level of Cx37 siRNA is negatively correlated with gastric cancer development and reduction of Cx37 expression promotes tumor cell apoptosis.

Expression of connexin subtypes are differentially regulated not only during metastasis but also during self-renewal of cancer cells. Among 131 cases of human astrocytic tumors, Cx43 was observed in 76.9 % of diffuse astrocytoma and in all cases of anaplastic astrocytoma and glioblastomas [125]. Cx46 was also detected in glioblastoma cancer stem cells, while Cx43 is predominantly expressed in non-stem cells [126]. Interestingly, during differentiation, Cx46 is reduced and Cx43 is increased, and targeting Cx46 reduces stem cell maintenance. Furthermore, by analyzing connexin subtype expression data of 1809 and 1899 breast cancer from Affymetrix and illumine arrays, respectively as well as 127 patients with all tumor grades, a correlation was established; elevated Cx43 protein levels were linked with improved breast cancer outcome while elevated Cx30 levels were associated with a reduced disease outcome [127]. Thus, Cx43 and Cx30 were suggested as potential independent prognostic markers for breast cancer. Cx30 gene was deleted in 25.8 % of 751 and mutated in 15 % of 158 gliomas and protein was absent in 28.9 % of 145 tumors [128]. However, consistent with Cx30 in breast cancer, Cx43 level is inversely correlated to survival in 2 cohorts of glioblastoma patients. Cx43 was also found to protect glioblastoma cells against radiation-induced DNA damage. The correlation of expression of adhesion molecules and connexin subtypes is also investigated in human colorectal cancer; a positive correlation between Cx26 and Cx32, but not Cx43 and adhesive proteins in patients without lymph node metastases while a positive association between Cx43 and E-cadherin with lymph node metastases [129]. Cx43 expression is correlated with oral squamous cell carcinoma and the overall survival time, and is proposed as an independent prognostic biomarker [130]. In another study, connexin expression was evaluated in 96 breast cancer patients before and after neoadjuvant chemotherapy [131]. Cx32 is positively correlated with HER2 expression pre-chemotherapy and with Ki67, a cell proliferation marker, post-chemotherapy, while Cx46 is negatively correlated with Ki67 and positively with better survival in both pre- and post-chemotherapy groups, suggesting that Cx26 and Cx46 could be utilized to assess prognosis and therapeutic outcomes. Reduction of Cx43 level is also suggested as a reliable marker for the prediction of cisplatin-based chemotherapy outcome and prognosis for patients with advanced non-small cell lung cancer [132]. Cx43 expression is also reported to correlate with early tumor recurrence, disease-free survival and overall survival in hepatitis B virus (HBV)-related hepatocellular carcinoma (HCC), implying Cx43 may delay early HCC recurrence, metastasis and poor prognosis after radical hepatectomy [133]. Therefore, understanding the specific expression pattern of connexins in various tumors could help establish new prognostic biomarkers and offer targeted, specific options for cancer treatment.

Drug resistance is a major challenge for cancer treatment. Cisplatin is a commonly used chemotherapeutic agents for advanced non-small cell lung cancer, but prolonged treatment acquires resistant due to development of EMT [134]. Overexpression of Cx43 reverses EMT and cisplatin resistance whereas Cx43 deletion initiates EMT and drug resistance. Patients with glioblastoma multiforme (GBM), an aggressive adult primary brain tumor with poor prognosis, develop resistance to the frontline chemotherapy, temozolomide (TMZ) a chemotherapeutic agent. In contrast to the situation in lung cancer, Cx43 was increased with the formation of cell-cell communication in the resistant tumor cells and this increase was induced by epidermal growth factor receptor (EGFR) activated JNK-ERK1/2-AP-1 signaling [135]. Another related study showed that Cx43 expression in human glioma cells enhances resistance to TMZ via a mitochondrial apoptosis pathway by the reduction in Bax/Bcl-2 ratio and the release of cytochrome C [136]. These studies suggest that reduction of Cx43 could help sensitize resistant cells to the chemotherapy.

Recently, a new paradigm was proposed by Colin Green’s laboratory based on the data obtained in chronic inflammatory disorders and trauma in the eye that protecting, maintaining or restoring cancer vasculature leads to reduced tumor hypoxia and promotes the survival of normal cells [137]. Given that Cx43 hemichannels are involved in vascular leakage and endothelial cell death [138], modulation of these channels may provide an alternative for cancer treatment. Together, with advanced understanding of the mechanism of connexin and connexin channels in various types and stages of cancer development and metastasis, drugs targeting connexins in cancer cells or/and host normal cells in next phase of cancer therapy are getting closer to become reality.

## Pannexin channels in cancer

There are several examples of human diseases that are either directly or indirectly linked to pannexins, in particular, Panx1. This is not that surprising given that pannexins are involved in taste sensation, airway defense, viral infection (HIV), the immune response, ischemic cell death, seizures, apoptosis and the differentiation of keratinocytes, chondrocytes, and neurons [139, 140]. Pannexins are also present and play an important role in the development of several tissues and cell types [139–142] and have been implicated in cell proliferation [11, 23, 143], differentiation [144], and inflammation [32]. Collectively, trends in pannexin links to disease support the notion that reduced levels of pannexin channels can be protective against disease onset and progression. For instance, in adult mice Panx1 expression has been shown to have a deleterious effect in neurodegeneration [145], ischemia [146–148], epileptic seizures [149, 150], and Crohn’s disease [151]. It is therefore possible that Panx1 expression may be needed at early stages of development, but needs to be down-regulated in the adult to avoid the negative effects of its expression in pathological states including cancer.

## Pannexin 1 upregulation or gain-of-function in cancer

Our current knowledge of Panx1 function in cancer is limited (see Table 2). However, the majority of published reports, with three notable exceptions [3, 152, 153] indicate that amplification or upregulation of Panx1 is prevalent in cancer cell lines and tumors compared to normal tissues. In some cases, the knockdown of Panx1 can revert the cancer cells to a more normal phenotype, as we reported for B16-BL6 mouse melanoma cells, described in detail below [154]. Other examples include the human glioma cell line U87-MG that had a significant reduction in proliferation upon Panx1 siRNA treatment [155]. Similarly, higher expression of Panx1 is present in human leukemic lymphocytes than in normal T cells [156]. At the transcript level, Panx1 over-expression was linked to higher metastatic spread in syngeneic hepatocarcinoma cell lines of different metastatic abilities based on microarray analysis [157]. Similarly, amplification and over-expression of Panx1 was reported in aggressive multiple myeloma cell lines [158]. In tissues, upregulation or amplification of *PANX1* was reported in human biopsies from most cancer types according to the analysis of cross-cancer alterations in the 89 cancer studies included on the cBioPortal.org for Cancer Genomics [159, 160]. However, the Cancer Genome Atlas also reports some examples of *PANX1* deletions present in human biopsies, indicating that Panx1 expression could be regulated differently in specific cancer types. At the protein level, Panx1 expression is listed in 17 out of 20 tumour types (high in colorectal, lung, urothelial and stomach cancers), and up to 70 % of human melanoma tumours show high levels of Panx1 protein (Human Protein Atlas [161]).

**Table 2:** Summary of the current literature on the role of pannexin channels in different cancer types

Pannexin	Cancer type	Description	Reference
Panx1	Breast	Gain of function mutation in *PANX1* increases metastasis	[162]
Panx1	Melanoma	Upregulated expression. Knockdown induces re-differentiation	[33, 154]
Panx1	Glioma	Tumour suppressor when overexpressed in Panx1-deficient rat C6 glioma cells, but endogenously expressed in human glioma cell lines. Accelerates glioma tumor aggregate formation	[153, 181]
Panx1	Glioma	Panx1 siRNA reduces proliferation of U87-MG human glioma cells	[155]
Panx1	Hepatocellular carcinoma	Upregulation linked to cancer spread in aggressive Hca-F cell lines	[157]
Panx1	Multiple myeloma	Gene amplification and upregulation in myeloma cell lines	[158]
Panx1	Colon	Interaction with Liver X receptor β for pyroptotic cell death	[178]
Panx1	Leukemia Osteosarcoma	Upregulated in leukemic cells compare to T-cells. Activated by chemotherapy drugs and immunogenic therapy to release ATP	[156, 186]
Panx1	Prostate	Bystander cell killing effect of TMPK/AZT	[185]
Panx1	Gall bladder adenocarcinoma	Downregulated expression in tumours	[3]
Panx2	Glioma	Tumour suppressor overexpressed in C6 glioma cells. Downregulated in human tumours and positive correlation with patient survival	[179, 183]
Panx2	Hepatocellular carcinoma	Potential tumour suppressor. Methylated in cancer samples	[184]
Panx2	Neuroblastoma	Knockdown in Neuro2a cells increases differentiation	[21]
Panx1, Panx3	Basal and squamous skin cell carcinoma	Downregulated in BCC and SCC tumours compared to normal skin	[152]

The most recent report of the role of Panx1 in cancer was published by Furlow et al. in the journal of *Nature Cell Biology* [162]. In this study, Panx1 was found to be expressed in many breast cancer cell lines, and a truncated mutant form of Panx1 was particularly enriched in highly metastatic breast cancer lines like CN-LM1A and MDA-LM2. The mutation was not found in the less aggressive parental cell lines CN34 and MDA-MB-231. This mutation (*C268T*) results in a truncated protein Panx1^1–89^that only contains the N-terminus, first trans-membrane domain and part of the first extracellular loop of Panx1. This mutant form is not functional on its own but when co-expressed with full-length Panx1 has a gain-of function effect on channel activity [162]. Interestingly, this same mutation had been previously reported in the Cancer Genome Atlas (TCGA) as K91del in patient biopsies from invasive breast carcinoma [159, 160]. Furlow et al. elegantly showed that breast cancer cells expressing the mutant Panx1, increase ATP release in vitro and in vivo under conditions of membrane stretch where the mechano-sensitive Panx1 channel is activated. They propose that this type of activation may occur due to mechanical deformation of the cells in the microvasculature of secondary organs during metastasis. Cells that express the mutant Panx1 have a survival advantage in metastatic progression since the ATP released acts on purinergic receptors (P2Y) to suppress apoptosis and reduce cell death in those tight spots of the microvasculature. This study not only described a clear role for Panx1 in cancer metastasis, but also highlighted the possibility of using pharmacological inhibition of the Panx1 channel in mouse models with known channel blockers such as Carbenoxolone as a new therapeutic option to reduce breast cancer metastasis [162].

## Panx1 in melanoma and links to inflammation

Melanomas account for 80 % of all skin cancer-related deaths [163], and in spite of recent progress with targeted and immuno-therapies [164] such as BRAF/MEK/ERK/c-KIT inhibitors, with limited success in long term disease control [165], there is still a desperate need for effective treatment options for this highly metastatic disease [166]. We have previously shown that mouse melanocytes have low Panx1 expression while the aggressiveness of isogenic melanoma cell lines [167] (B16-FO, F10 and BL6) is directly correlated to their Panx1 levels, with no detectable Panx2 or Panx3 expression [154]. Knocking down Panx1 with shRNA in aggressive BL6 cells, changed cell morphology into a normal melanocytic phenotype, increased differentiation and melanin production, reduced migration and proliferation and affected the expression of malignant markers such as vimentin [154, 168]. In a chick-chorioallantoic membrane assay [169–172] (chick-CAM), the mouse melanoma tumors with Panx1 shRNA were significantly smaller and had less instances of metastasis to the liver than the aggressive wild-type cells with high Panx1 [154]. We proposed Panx1 as an interesting new target for therapeutic intervention in the fight against melanoma.

Recently, two articles have highlighted the link between inflammation and melanoma, proposing the involvement of the Panx1/P2X7 complex as a key regulator [33, 173]. The Panx1/P2X7 complex has been shown to activate the NLRP3 inflammasome and participate in the release of IL-1β in vitro [34, 174, 175]. It is known that the NLRP3 inflammasome is constitutively activated in melanoma cells, and late-stage melanoma cells can autonomously secrete IL-1β [176]. IL-1β is a pro-inflammatory cytokine involved in many processes including cell proliferation, tissue regeneration and immune regulation. It is also a tumor promoting factor implicated in cancer progression, increasing angiogenesis, invasion and metastasis (as reviewed by [177]). Additionally, IL-1β may also drive expression of cyclooxygenase-2 (COX2) which leads to high levels of prostaglandin E_2_ [33]. In the proposed model, ATP activates the Panx1/P2X7 complex to open (in a mutated melanocytic cell) and allows the cation flow that activates the NLRP3 inflammasome composed of NLR (nucleotide oligomerization domain-like, leucine-rich repeat containing receptor), ASC (apoptosis associated speck-like protein containing a caspase recruitment domain (CARD)), and caspase-1. Activated caspase-1 cleaves and activates the cytokines (IL-18 and IL-1β). IL-1 can then increase expression of COX-2 and PGE_2_. Both IL-1β and PGE_2_ can be released from the cell to act on the microenvironment. The effects would include increased cell proliferation, angiogenesis, tumor invasiveness and growth, and recruitment of pro-inflammatory cells. Other mutations in the melanoma cell alter normal growth cycle feedback, and with this inflammasome activation, may continue to proliferate without control [33]. A caveat to this model is the possibility that high levels of ATP may do the opposite and reduce growth and tumorigenicity in cancer cells as pointed out by Mantel and Harvey [173]. Additionally, this same pathway involving the NLRP3 inflammasome, can result in the activation of cell death and pyroptosis, which would have the opposite effect. This alternative route of the NLRP3 signalling model was shown recently to also involve Panx1 [178]. In colon cancer cells that express liver X receptors (LXRs), P2X7 receptors and Panx1, it was proposed that the LXR ligand would bind its receptor and facilitate LXR’s association with Panx1. This new complex allows the opening of Panx1 channels to release ATP for autocrine activation of P2X7 receptors. The P2X7 activation then leads to NLRP3 inflammasome assembly, and activated caspase-1 that induces colon cancer cell pyroptosis and death [178]. In conclusion, Panx1/P2X7 is upstream of the NLRP3 inflammasome, but two different arms of its downstream signalling pathway could be either implicated in tumorigenesis in the case of melanomas, or cell death in the case of colon cancer cells that express LXRs.

## Downregulation of Pannexin expression in cancer

As was mentioned before, even though the majority of reports in the literature to date point towards an over-expression or amplification of Panx1 in cancer cells and tumours, there are also important exceptions to this trend. In fact, one of the first papers involving Panx1 and cancer reported that while Panx1 mRNA was expressed in primary astrocytes, it was not expressed in the C6 rat glioma cell line. Paradoxically, the authors reported four human glioma cell lines (U87, U251, SF188 and SF539) that were all positive for Panx1 at the transcript level, indicating that there might be some differences among species in terms of Panx1 expression in gliomas. Additionally, when Panx1-GFP was overexpressed in C6 glioma cells [153, 179], it appeared to have a tumour suppressive effect. These latter findings are complicated to interpret as we now know that the GFP tag has a negative effect on the Panx1 channel function and trafficking [180]. However, it is possible that pannexins may have different roles in different cancer subtypes, as pointed out in the previous section where the co-expression of additional receptors (such as LXR) may affect the downstream signaling consequences and result in different cellular outcomes. Interestingly, these C6 glioma cells stably expressing Panx1-GFP were also used to demonstrate in a later report, that Panx1 expression can accelerate the assembly of multicellular tumor aggregates through an interaction with F-actin microfilaments. The formation of these large cellular aggregates is a key step in glioma progression [181].

A second case of downregulation of Panx1 in cancer tissues was reported in a review paper by Schalper et al. [3] where immunohistochemistry of normal human gallbladder and gallbladder adenocarcinoma with anti- Panx1 antibodies indicated a lower expression of Panx1 in tumour cells. Additionally, using Ki67 labeling along with Panx1 immunostaining, the authors concluded that Panx1 expression was negatively correlated with proliferation in gallbladder carcinomas [3].

The third example of downregulation of pannexins in tumours was reported in non-melanoma skin cancer samples from human biopsies by our collaborators [152]. While Panx1 and Panx3 are expressed in normal adult human facial skin, their expression is not detectable in either basal or squamous cell carcinomas immunostained with anti-pannexin antibodies. This apparent downregulation of Panx1 and 3 in keratinocytic tumours may indicate a protective role of these pannexins against keratinocyte transformation [152]. However, it is also possible that cells that originally express pannexins in these type of tumours, may go through the keratinocyte life cycle at a higher rate to proliferate, differentiate and undergo programmed cell death, and are therefore not detectable in advanced skin cancer biopsies.

## Pannexin 2 as a tumour suppressor

In the pannexin family, Panx2 and Panx3 are not as well characterized as their more popular and ubiquitously expressed sibling. Therefore, our understanding of the role of these two isoforms in cancer is limited. Panx3 plays a role in proliferation and differentiation of cells in skin, cartilage and bone [182], so we predict that we will soon learn more about a potential role for Panx3 in skin cancer and osteosarcomas. Panx2 has been proposed to play an important role as a tumour suppressor in brain tumours and hepatocellular carcinomas (Table 2).

Litvin et al. was the first to publish the initial evidence of a down-regulation of human Panx2 transcripts in Affymetrix chip analysis, particularly in astrocytomas, glioblastoma multiforme (GBM), and oligodendro-gliomas [183]. Additionally, in Kaplan-Meier survival plots of differential expression of Panx2 in human tumour samples, there was a positive correlation between Panx2 expression and patient survival [183].

Lai et al. [179] described the downregulation of Panx2 mRNA in C6 glioma cells, which was consistent with a reduction or absence of expression in human glioblastoma, glioma and GBM cell lines at the transcript level compared to normal astrocytes and human brain. Importantly, the over-expression of Panx2-GFP or Panx2-HA in C6 cells changed cell morphology, reduced cell growth and anchorage-independent growth. Over-expressed Panx2-HA or -GFP tagged proteins had a significant reduction in in vivo tumour growth in nude mice when compared to controls [179].

Consistent with a potential role as tumour suppressor, a recent publication listed *PANX2* as one of the methylated genes identified by expression profiling and DNA methylation arrays in hepatocellular carcinomas (HCC) [184]. In HCC, the hepatocyte growth factor (HGF) upregulates the expression of DNA methyltransferase 1 that results in the hypermethylation and epigenetic repression of potential tumour suppressor genes, including *PANX2*. Recently, Le Vasseur et al. demonstrated that Panx2 expression is not limited to the central nervous system as was originally proposed, and it was shown to be expressed in liver among many other organs and tissues [20]. Therefore, it is possible that Panx2 expression is silenced upon transformation in many tissues.

## Chemotherapeutic agents and Panx1 channels

As we discussed earlier, Panx1 is commonly expressed and even upregulated in many cancer cells. An unexpected side effect of this Panx1 expression, other than its potential role in cancer progression, is the possibility that Panx1 channels in the presence of different chemotherapeutic drugs, can actually be exploited to enhance cancer cell death. One example of this was seen in prostate cancer PC-3 cells engineered by gene therapy to produce a variant of human thymidylate kinase (TMPK) that can potentiate the drug azido thymidine (AZT). Panx1 is necessary for this effective bystander cell killing of neighbouring cells after suicide gene therapy delivered by lentivirus [185]. However, the authors propose that this effect is the result of Panx1 gap junctions between cells, and the effect is abolished by treatment with carbenoxolone. In this case, since the cells express high levels of Cx43, the observed effect may be due to real connexin gap junctions, even if not detected as plaques at the cell surface as was expected by the authors [185].

Some more clear examples of the role of Panx1 channels in the presence of chemotherapeutic drugs were reported by Boyd-Tressler et al. [156] and Martins et al. [186]. The first group showed upregulation of Panx1 in leukemic cells compared to normal T-cells. In Jurkat cells they observed that in the presence of different chemotherapy drugs that induce apoptosis, Panx1 channels were cleaved by caspase-3 to remove the C-tail inhibitory fragment and were activated to open and release ATP, ADP and AMP. These nucleotides can also be released by other Panx1-independent mechanisms, and are very important as paracrine signals to effector leukocytes that mediate the immune response against the dying tumor cells [156]. A similar effect was observed in Panx1-expressing human osteosarcoma cells (U2OS) and fibrosarcomas (MCA205) that were challenged with immunogenic cell death- inducing antineoplastic agents (oxaliplatin, anthracyclines) [186]. During cell death, the release of ATP happens in this case, through a different mechanism that involves lysosomal exocytosis. The effect of caspases and the opening of Panx1 channels is required for the translocation of LAMP1 to the cell surface for ATP release [186].

It is encouraging to think that Panx1 could be used as a biomarker in different cancers, but also as a conduit of ATP that assists the process of cancer cell death by existing chemotherapies, and facilitates immune recognition of the targeted tumour.

## Panx1 channel blockers and their potential therapeutic use in cancer

There are now several compounds that have been reported in the literature to efficiently block Panx1 channels that could be used as new therapeutic alternatives. Namely, probenecid (PBN) [180, 187, 188], carbenoxolone [189, 190], trovafloxaxin (Trova) [191], and mefloquine (MFQ) [192] have been successfully used in vitro and/or in vivo to close Panx1 channels with no reported significant side effects. However, as with any chemical blocker, there are also many unspecific effects reported that should be taken into account. At present PBN (used in the past for long-term treatment of gout as it interferes with the organic anion transporter) is frequently used as Panx1 channel inhibitor [180, 187]. In fact, PBN has been recently used as an effective adjuvant therapy to sensitize breast cancer cells and enhance bisphosphonate chemotherapy anti-tumor effects [193]. CBX has also been shown to be specific to Panx1 in lower doses, because at higher doses (>100 μM) it may also block connexin hemichannels [180, 187]. MFQ (a malaria drug), has been used successfully in mice to block Panx1 [192, 194–196]. Trova is an antibiotic (toxic in high doses) that has been recently reported to specifically block Panx1 as well [191]. In addition to these compounds, there are other small molecule inhibitors such as the ^10^Panx1 peptide [147, 197] and specific anti-Panx1 antibodies [148] that have also been widely tested for their inhibitory effects on Panx1 channel function. These are tools that could be exploited as different ways of inhibiting the Panx1 channel that may prove useful as novel treatments in the fight against different cancers.

## Conclusions

Cancer is not a single disease but a term that includes many different and complex diseases. The role of cellular communication in cancer, facilitated by the connexin and pannexin families of channel-forming proteins is also of a complex nature. There are 21 connexins and 3 pannexins in the human genome, making it challenging to generalize about their overall function in cancer. In very general terms, the current literature would support a tumour suppressor role for connexins in early stages of cancer progression, with an opposing role in late-stage (or advanced) cancer and metastasis. On the pannexin side, the majority of reports point towards a tumour promoting effect of Panx1 expression. However, this effect may be different for the other pannexin members, and may also be tissue- and cancer- specific. Our developing knowledge of the mechanisms that regulate gap junctions, connexin hemichannels, and pannexin channels in the different cancer types will allow us to better target connexins and pannexins as new candidates for therapeutic intervention.
